# 2,6-Bis(2-chloro­phen­yl)-4-oxo-3,5-diphenyl­heptane-1,1,7,7-tetra­carbo­nitrile

**DOI:** 10.1107/S1600536811017284

**Published:** 2011-05-14

**Authors:** A. Jahubar Ali, S. Athimoolam, S. Asath Bahadur, V. P. Alex Raja

**Affiliations:** a Department of Science and Humanities, National College of Engineering, Maruthakulam, Tirunelveli 627 151, India; b Department of Physics, University College of Engineering Nagercoil, Anna University of Technology Tirunelveli, Nagercoil 629 004, India; c Department of Physics, Kalasalingam University, Anand Nagar, Krishnan Koil 626 190, India; d Department of Organic Chemistry, Madurai Kamaraj University, Madurai 625 021, India

## Abstract

In the title compound, C_35_H_24_Cl_2_N_4_O, the phenyl rings are oriented almost parallel to each other, making a dihedral angle of 0.6 (2)°, whereas the chloro­phenyl rings are oriented at a dihedral angle of 28.3 (1)°. The crystal structure is stabilized through an extensive series of C—H⋯N, C—H⋯O and C—H⋯Cl inter­actions. One of the C—H⋯N inter­actions generates an *R*_2_^2^(12) ring motif around a crystallographic inversion centre. *C*(5), *C*(10) and *C*(12) chain motifs are observed in the unit cell through C—H⋯N and C—H⋯Cl inter­actions. During the structure analysis, it was observed that the unit cell contains large accessible voids, which host disordered solvent mol­ecules. This affects the diffraction pattern, mostly at low scattering angles and was corrected with the *SQUEEZE* program [Spek, A. L. (2009 ▶). *Acta Cryst.* D**65**, 148–155].

## Related literature

For our investigations into regio/stereoselectivity in adduct reactions and weak hydrogen bonding, see: Ali *et al.* (2010 ▶). For weak hydrogen-bonding inter­actions, see: Desiraju & Steiner (1999 ▶). For ring and chain motifs, see: Etter *et al.* (1990 ▶).
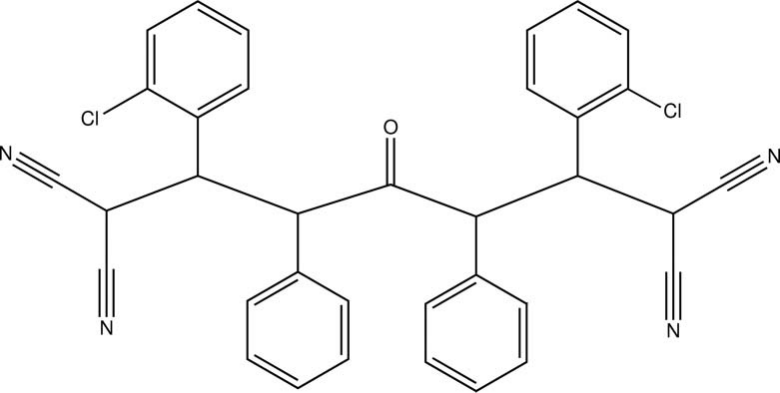


## Experimental

### 

#### Crystal data

C_35_H_24_Cl_2_N_4_O
*M* *_r_* = 587.48
Monoclinic, 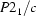

*a* = 17.7226 (6) Å
*b* = 10.6169 (3) Å
*c* = 20.8491 (7) Å
β = 113.724 (2)°
*V* = 3591.4 (2) Å^3^
*Z* = 4
Mo *K*α radiation
μ = 0.21 mm^−1^
*T* = 293 K
0.28 × 0.26 × 0.22 mm

#### Data collection

Bruker SMART APEX CCD area-detector diffractometer
32501 measured reflections
6324 independent reflections
3647 reflections with *I* > 2σ(*I*)
*R*_int_ = 0.042

#### Refinement

*R*[*F* ^2^ > 2σ(*F* ^2^)] = 0.061
*wR*(*F* ^2^) = 0.211
*S* = 1.07
6324 reflections
379 parameters
H-atom parameters constrained
Δρ_max_ = 0.31 e Å^−3^
Δρ_min_ = −0.27 e Å^−3^

### 

Data collection: *SMART* (Bruker, 2001 ▶); cell refinement: *SAINT* (Bruker, 2001 ▶); data reduction: *SAINT*; program(s) used to solve structure: *SHELXTL/PC* (Sheldrick, 2008 ▶); program(s) used to refine structure: *SHELXTL/PC*; molecular graphics: *Mercury* (Macrae *et al.*, 2006 ▶) and *PLATON* (Spek, 2009 ▶); software used to prepare material for publication: *SHELXTL/PC*.

## Supplementary Material

Crystal structure: contains datablocks global, I. DOI: 10.1107/S1600536811017284/sj5139sup1.cif

Structure factors: contains datablocks I. DOI: 10.1107/S1600536811017284/sj5139Isup2.hkl

Supplementary material file. DOI: 10.1107/S1600536811017284/sj5139Isup3.cml

Additional supplementary materials:  crystallographic information; 3D view; checkCIF report

## Figures and Tables

**Table 1:** Hydrogen-bond geometry (Å, °)

*D*—H⋯*A*	*D*—H	H⋯*A*	*D*⋯*A*	*D*—H⋯*A*
C7—H7⋯N11^i^	0.98	2.33	3.186 (4)	145
C23—H23⋯N11^ii^	0.93	2.65	3.409 (5)	139
C34—H34⋯N72^iii^	0.93	2.52	3.443 (8)	176
C52—H52⋯N12^iv^	0.93	2.64	3.489 (5)	152
C36—H36⋯N12^iv^	0.93	2.96	3.805 (7)	152
C54—H54⋯N71^v^	0.93	2.91	3.564 (6)	128
C53—H53⋯N71^v^	0.93	2.96	3.583 (5)	126
C64—H64⋯Cl2^vi^	0.93	2.97	3.663 (5)	133
C64—H64⋯O1^vi^	0.93	2.88	3.673 (5)	144
C65—H65⋯Cl1^vi^	0.93	2.80	3.728 (4)	174

Symmetry codes: (i) 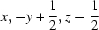; (ii) 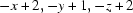; (iii) 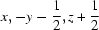; (iv) 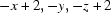; (v) 
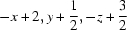
; (vi) 
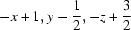
.

**Fig. 1.**
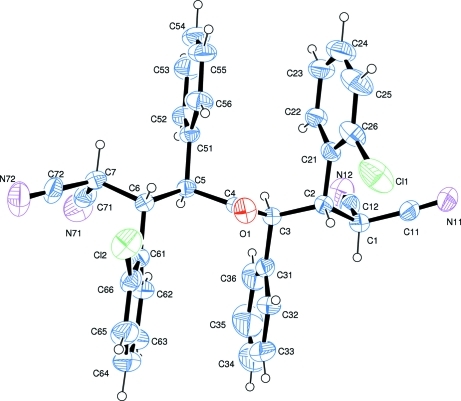
The title molecule with the atom numbering scheme. The displacement ellipsoids are shown at the 30% probability level.

**Fig. 2.**
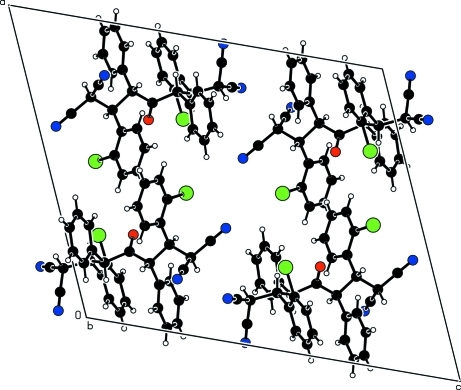
Packing diagram of the title structure viewed down the a axis. (Cl is shown in green, N in blue, O in red, C in black and H as a circles)

**Fig. 3.**
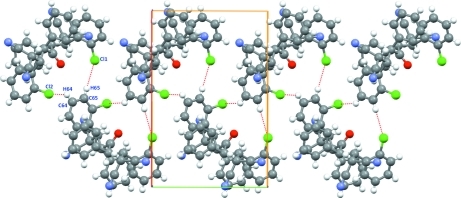
Linear chain C(5) and zigzag chain C(12) motifs speckled along ab-plane of the crystal through C—H···Cl interactions. (Cl is shown in green, N in blue, O in red, C in black and H as a circles)

**Fig. 4.**
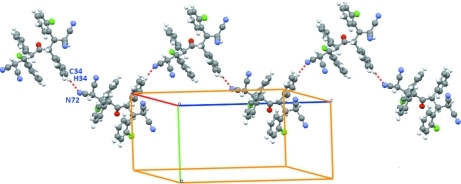
Zigzag chain C(12) motif extending along c axis of the unit cell. (Cl is shown in green, N in blue, O in red, C in black and H as a circles)

**Fig. 5.**
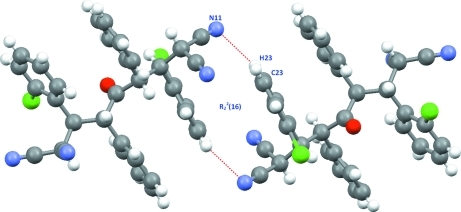
Ring R22(12) motif formed through C—H···N interactions. (Cl is shown in green, N in blue, O in red, C in black and H as a circles)

## supplementary crystallographic information

### Comment

In continuation of our investigations into regio/stereoselectivity in adduct reactions and weak hydrogen bonding (Ali *et al.*, 2010), the title compound was synthesized and crystallized and the structural features are disucussed here.

The molecular structure of the title compound is shown in Fig. 1. The two phenyl rings are oriented almost parallel to each other with the dihedral angle of 0.6 (2)°, whereas the two chlorophenyl rings are oriented with an angle of 28.3 (1)°. This large variation may be due to the strong and moderate C—H···Cl interactions observed in the lattice. Also, due to these C—H···Cl interactions in the crystal packing (Fig. 2, Table 1), the chlorine atoms in the chlorophenyl rings lie away from the benzene ring planes with the distances of 0.01 (1)Å (for Cl1 atom in C21/C26/Cl1 ring) and 0.08 (1)Å (for Cl2 atom in C61/C66/Cl2 ring).

The packing diagram of the title compound is shown in Fig. 2. The crystal packing is stabilized through an extensive series of C—H···N, C—H···O and C—H···Cl interactions (Desiraju & Steiner, 1999). One C—H···O, two C—H···Cl and seven C—H···N interactions are observed in the lattice (Table 1). The two C—H···Cl interactions are involved in making chain motifs, *viz*., a zigzag C(12) chain motif [through C65—H65···Cl1 (-*x* + 1, *y* - 1/2,-*z* + 3/2)] and a linear C(5) chain motif [through C64—H64···Cl2 (-*x* + 1, *y* - 1/2, -*z* + 3/2)] (Etter *et al.*, 1990). These chain motifs are speckled on the *ab*-plane of the unit cell as shown in Fig. 3. A C(10) chain motif is observed through C7—H7···N11 (*x*, -*y* + 1/2, 2–1/2) interactions which connect the molecules in a head-to-tail fashion along the *c* axis. Another C—H···N interaction makes a zigzag C(12) chain motif extending along *c* (Fig. 4). A centrosymmetric *R*_2_^2^(16) ring motif is observed around a crystallographic inversion centre through C—H···N interactions (Fig. 5).

### Experimental

A mixture of 1,3-diphenylacetone 5 (1 mmol), 2-[(2-chlorophenyl)methylene]malononitrile 6 (2 mmol), and sodium ethoxide (2 mmol) was ground well in a mortar and pestle at ambient temperature for about 15–30 sec. Then water (50–70 ml) was added to the mixture and the product was filtered and washed with water, dried *in vacuo* and subjected to flash chromatographic purification employing flash silica gel (230–400 mesh) with petroleum ether-ethyl acetate mixture (1:2 *v*/*v*) as eluent. The products were further recrystallized from ethanol-ethyl acetate mixture (1:2 *v*/*v*).

### Refinement

All the H atoms were positioned geometrically and refined by the riding model approximation with d(C—H) = 0.93 - 0.98 Å and i>U_iso_(H)= 1.2U_eq_(C).  During the structure analysis, it was observed that the unit cell contains large accessible voids in the crystal structure which tend to host unpredictable disordered  solvent molecules. This affects the diffraction pattern, mostly at low scattering angles and was corrected with the SQUEEZE program (Spek, 2009).

### Crystal data

**Table:**

C_35_H_24_Cl_2_N_4_O	*F*(000) = 1216
*M**_r_* = 587.48	*D*_x_ = 1.087 Mg m^−^^3^
Monoclinic, *P*2_1_/*c*	Mo *K*α radiation, λ = 0.71073 Å
Hall symbol: -P 2ybc	Cell parameters from 4531 reflections
*a* = 17.7226 (6) Å	θ = 2.8–24.8°
*b* = 10.6169 (3) Å	µ = 0.21 mm^−^^1^
*c* = 20.8491 (7) Å	*T* = 293 K
β = 113.724 (2)°	Bulk, colourless
*V* = 3591.4 (2) Å^3^	0.28 × 0.26 × 0.22 mm
*Z* = 4	

### Data collection

**Table:**

Bruker SMART APEX CCD area-detector diffractometer	3647 reflections with *I* > 2σ(*I*)
Radiation source: fine-focus sealed tube	*R*_int_ = 0.042
graphite	θ_max_ = 25.0°, θ_min_ = 1.3°
ω scans	*h* = −21→21
32501 measured reflections	*k* = −12→12
6324 independent reflections	*l* = −24→24

### Refinement

**Table:**

Refinement on *F*^2^	Primary atom site location: structure-invariant direct methods
Least-squares matrix: full	Secondary atom site location: difference Fourier map
*R*[*F*^2^ > 2σ(*F*^2^)] = 0.061	Hydrogen site location: inferred from neighbouring sites
*wR*(*F*^2^) = 0.211	H-atom parameters constrained
*S* = 1.07	*w* = 1/[σ^2^(*F*_o_^2^) + (0.114*P*)^2^ + 0.3187*P*] where *P* = (*F*_o_^2^ + 2*F*_c_^2^)/3
6324 reflections	(Δ/σ)_max_ < 0.001
379 parameters	Δρ_max_ = 0.31 e Å^−^^3^
0 restraints	Δρ_min_ = −0.27 e Å^−^^3^

### Special details

**Table:**

Geometry. All e.s.d.'s (except the e.s.d. in the dihedral angle between two l.s. planes) are estimated using the full covariance matrix. The cell e.s.d.'s are taken into account individually in the estimation of e.s.d.'s in distances, angles and torsion angles; correlations between e.s.d.'s in cell parameters are only used when they are defined by crystal symmetry. An approximate (isotropic) treatment of cell e.s.d.'s is used for estimating e.s.d.'s involving l.s. planes.
Refinement. Refinement of *F*^2^ against ALL reflections. The weighted *R*-factor *wR* and goodness of fit *S* are based on *F*^2^, conventional *R*-factors *R* are based on *F*, with *F* set to zero for negative *F*^2^. The threshold expression of *F*^2^ > σ(*F*^2^) is used only for calculating *R*-factors(gt) *etc*. and is not relevant to the choice of reflections for refinement. *R*-factors based on *F*^2^ are statistically about twice as large as those based on *F*, and *R*- factors based on ALL data will be even larger.

### Fractional atomic coordinates and isotropic or equivalent isotropic displacement parameters (Å^2^)

**Table:**

	*x*	*y*	*z*	*U*_iso_*/*U*_eq_	
C1	0.8486 (2)	0.2323 (3)	1.03039 (17)	0.0623 (9)	
H1	0.8131	0.1752	1.0428	0.075*	
C2	0.80856 (18)	0.2513 (2)	0.95012 (16)	0.0511 (7)	
H2	0.7513	0.2768	0.9379	0.061*	
C3	0.80630 (17)	0.1253 (2)	0.91167 (15)	0.0471 (7)	
H3	0.8628	0.1041	0.9185	0.057*	
C4	0.75542 (17)	0.1395 (2)	0.83391 (14)	0.0432 (7)	
C5	0.78235 (17)	0.0628 (2)	0.78571 (14)	0.0469 (7)	
H5	0.8066	−0.0159	0.8097	0.056*	
C6	0.70861 (17)	0.0294 (2)	0.71749 (15)	0.0478 (7)	
H6	0.6838	0.1091	0.6953	0.057*	
C7	0.73918 (19)	−0.0388 (3)	0.66625 (17)	0.0574 (8)	
H7	0.7774	0.0182	0.6574	0.069*	
C11	0.8527 (2)	0.3532 (4)	1.06706 (19)	0.0695 (9)	
C12	0.9297 (3)	0.1773 (3)	1.05569 (18)	0.0682 (9)	
C21	0.84917 (19)	0.3550 (3)	0.92648 (16)	0.0544 (8)	
C22	0.9254 (2)	0.3393 (3)	0.92435 (18)	0.0649 (9)	
H22	0.9522	0.2625	0.9386	0.078*	
C23	0.9631 (3)	0.4317 (4)	0.9022 (2)	0.0888 (12)	
H23	1.0151	0.4180	0.9025	0.107*	
C24	0.9248 (4)	0.5425 (5)	0.8801 (2)	0.1061 (16)	
H24	0.9499	0.6049	0.8641	0.127*	
C25	0.8508 (3)	0.5635 (4)	0.8809 (3)	0.1058 (15)	
H25	0.8248	0.6405	0.8654	0.127*	
C26	0.8118 (2)	0.4710 (3)	0.9049 (2)	0.0785 (11)	
C31	0.7717 (3)	0.0166 (3)	0.93763 (18)	0.0689 (10)	
C32	0.6897 (3)	0.0163 (4)	0.9284 (2)	0.0900 (13)	
H32	0.6555	0.0832	0.9059	0.108*	
C33	0.6594 (5)	−0.0833 (6)	0.9528 (4)	0.148 (3)	
H33	0.6047	−0.0834	0.9473	0.178*	
C34	0.7084 (9)	−0.1801 (8)	0.9843 (5)	0.189 (5)	
H34	0.6874	−0.2456	1.0018	0.227*	
C35	0.7875 (7)	−0.1860 (6)	0.9916 (4)	0.167 (3)	
H35	0.8196	−0.2565	1.0112	0.200*	
C36	0.8197 (4)	−0.0850 (3)	0.9692 (2)	0.1048 (15)	
H36	0.8747	−0.0861	0.9756	0.126*	
C51	0.84908 (17)	0.1359 (2)	0.77357 (15)	0.0483 (7)	
C52	0.9270 (2)	0.0907 (3)	0.79355 (18)	0.0672 (9)	
H52	0.9402	0.0123	0.8150	0.081*	
C53	0.9874 (2)	0.1595 (4)	0.7825 (2)	0.0877 (12)	
H53	1.0407	0.1282	0.7971	0.105*	
C54	0.9676 (3)	0.2725 (4)	0.7502 (2)	0.0889 (12)	
H54	1.0074	0.3184	0.7418	0.107*	
C55	0.8901 (3)	0.3199 (4)	0.7298 (2)	0.0827 (11)	
H55	0.8775	0.3978	0.7076	0.099*	
C56	0.8309 (2)	0.2548 (3)	0.74135 (19)	0.0692 (9)	
H56	0.7784	0.2888	0.7280	0.083*	
C61	0.64183 (18)	−0.0448 (2)	0.72805 (15)	0.0498 (7)	
C62	0.6602 (2)	−0.1490 (3)	0.77301 (18)	0.0629 (9)	
H62	0.7149	−0.1713	0.7984	0.076*	
C63	0.5993 (3)	−0.2186 (3)	0.7803 (2)	0.0818 (11)	
H63	0.6132	−0.2869	0.8108	0.098*	
C64	0.5187 (3)	−0.1890 (5)	0.7436 (3)	0.0953 (13)	
H64	0.4776	−0.2379	0.7482	0.114*	
C65	0.4985 (2)	−0.0889 (4)	0.7005 (2)	0.0857 (12)	
H65	0.4433	−0.0676	0.6764	0.103*	
C66	0.5586 (2)	−0.0174 (3)	0.69163 (17)	0.0598 (8)	
C71	0.7844 (2)	−0.1558 (4)	0.6959 (2)	0.0728 (10)	
C72	0.6731 (2)	−0.0659 (3)	0.5998 (2)	0.0707 (9)	
Cl1	0.71848 (7)	0.50340 (9)	0.90878 (9)	0.1247 (6)	
Cl2	0.52710 (6)	0.10805 (10)	0.63456 (5)	0.0895 (4)	
N11	0.8558 (2)	0.4487 (4)	1.09207 (19)	0.0953 (11)	
N12	0.9931 (3)	0.1336 (4)	1.0738 (2)	0.1026 (11)	
N71	0.8197 (2)	−0.2441 (4)	0.7193 (2)	0.1078 (12)	
N72	0.6215 (2)	−0.0886 (4)	0.5485 (2)	0.1075 (12)	
O1	0.69628 (13)	0.20891 (19)	0.81126 (12)	0.0644 (6)	

### Atomic displacement parameters (Å^2^)

**Table:**

	*U*^11^	*U*^22^	*U*^33^	*U*^12^	*U*^13^	*U*^23^
C1	0.073 (2)	0.0613 (17)	0.060 (2)	−0.0149 (17)	0.0352 (19)	−0.0043 (15)
C2	0.0488 (17)	0.0524 (15)	0.0531 (19)	−0.0042 (13)	0.0216 (15)	−0.0043 (13)
C3	0.0486 (17)	0.0468 (14)	0.0483 (18)	−0.0044 (12)	0.0220 (15)	−0.0012 (12)
C4	0.0418 (16)	0.0410 (13)	0.0476 (18)	−0.0076 (13)	0.0189 (14)	0.0009 (12)
C5	0.0474 (17)	0.0470 (14)	0.0462 (17)	0.0011 (12)	0.0189 (14)	0.0046 (12)
C6	0.0509 (17)	0.0503 (15)	0.0453 (17)	−0.0023 (13)	0.0228 (15)	0.0008 (12)
C7	0.0534 (19)	0.0652 (17)	0.064 (2)	−0.0067 (15)	0.0342 (18)	−0.0031 (15)
C11	0.065 (2)	0.087 (2)	0.067 (2)	−0.0161 (18)	0.0382 (19)	−0.0177 (19)
C12	0.079 (3)	0.073 (2)	0.049 (2)	−0.005 (2)	0.020 (2)	−0.0031 (16)
C21	0.057 (2)	0.0517 (16)	0.0488 (18)	−0.0103 (14)	0.0153 (15)	−0.0030 (13)
C22	0.065 (2)	0.0675 (19)	0.062 (2)	−0.0153 (16)	0.0255 (18)	−0.0023 (16)
C23	0.090 (3)	0.098 (3)	0.083 (3)	−0.038 (2)	0.039 (2)	−0.002 (2)
C24	0.131 (4)	0.095 (3)	0.086 (3)	−0.053 (3)	0.037 (3)	0.007 (2)
C25	0.116 (4)	0.059 (2)	0.119 (4)	−0.014 (2)	0.023 (3)	0.023 (2)
C26	0.072 (2)	0.0543 (18)	0.092 (3)	−0.0060 (17)	0.015 (2)	0.0025 (17)
C31	0.105 (3)	0.0566 (18)	0.057 (2)	−0.0177 (18)	0.045 (2)	−0.0049 (15)
C32	0.104 (3)	0.084 (2)	0.110 (3)	−0.040 (2)	0.072 (3)	−0.023 (2)
C33	0.227 (7)	0.122 (4)	0.172 (6)	−0.084 (5)	0.159 (6)	−0.050 (4)
C34	0.367 (15)	0.104 (5)	0.152 (6)	−0.095 (8)	0.163 (9)	−0.018 (4)
C35	0.291 (10)	0.073 (3)	0.151 (6)	−0.013 (5)	0.104 (7)	0.023 (3)
C36	0.169 (5)	0.054 (2)	0.096 (3)	0.003 (2)	0.058 (3)	0.017 (2)
C51	0.0420 (17)	0.0577 (16)	0.0450 (17)	−0.0033 (13)	0.0173 (14)	0.0051 (13)
C52	0.055 (2)	0.075 (2)	0.076 (2)	0.0024 (16)	0.0301 (19)	0.0075 (17)
C53	0.055 (2)	0.110 (3)	0.105 (3)	−0.007 (2)	0.039 (2)	−0.004 (3)
C54	0.071 (3)	0.114 (3)	0.090 (3)	−0.025 (2)	0.042 (2)	0.006 (2)
C55	0.083 (3)	0.088 (2)	0.076 (3)	−0.021 (2)	0.031 (2)	0.021 (2)
C56	0.057 (2)	0.0697 (19)	0.073 (2)	−0.0032 (16)	0.0188 (18)	0.0171 (17)
C61	0.0455 (17)	0.0542 (15)	0.0548 (19)	−0.0018 (13)	0.0257 (15)	−0.0092 (14)
C62	0.060 (2)	0.0599 (17)	0.072 (2)	−0.0060 (15)	0.0300 (18)	0.0092 (16)
C63	0.083 (3)	0.076 (2)	0.095 (3)	−0.016 (2)	0.044 (2)	0.001 (2)
C64	0.084 (3)	0.118 (3)	0.097 (3)	−0.034 (3)	0.050 (3)	−0.007 (3)
C65	0.046 (2)	0.129 (3)	0.085 (3)	−0.004 (2)	0.030 (2)	−0.008 (3)
C66	0.050 (2)	0.078 (2)	0.054 (2)	0.0052 (16)	0.0242 (16)	−0.0077 (15)
C71	0.066 (2)	0.088 (2)	0.075 (3)	0.006 (2)	0.040 (2)	−0.007 (2)
C72	0.068 (2)	0.092 (2)	0.056 (2)	0.005 (2)	0.029 (2)	−0.0180 (19)
Cl1	0.0854 (8)	0.0705 (6)	0.1940 (15)	0.0109 (5)	0.0308 (9)	−0.0038 (7)
Cl2	0.0723 (7)	0.1123 (8)	0.0810 (7)	0.0277 (5)	0.0279 (5)	0.0140 (5)
N11	0.100 (3)	0.107 (2)	0.104 (3)	−0.024 (2)	0.067 (2)	−0.040 (2)
N12	0.106 (3)	0.112 (3)	0.081 (3)	0.016 (2)	0.028 (2)	0.007 (2)
N71	0.100 (3)	0.104 (3)	0.128 (3)	0.034 (2)	0.055 (3)	0.010 (2)
N72	0.089 (3)	0.141 (3)	0.083 (3)	0.001 (2)	0.026 (2)	−0.041 (2)
O1	0.0532 (13)	0.0653 (12)	0.0664 (15)	0.0092 (11)	0.0154 (11)	−0.0047 (11)

### Geometric parameters (Å, °)

**Table:**

C1—C12	1.441 (5)	C31—C32	1.387 (5)
C1—C11	1.481 (5)	C32—C33	1.373 (6)
C1—C2	1.546 (4)	C32—H32	0.9300
C1—H1	0.9800	C33—C34	1.335 (12)
C2—C21	1.503 (4)	C33—H33	0.9300
C2—C3	1.551 (4)	C34—C35	1.348 (12)
C2—H2	0.9800	C34—H34	0.9300
C3—C31	1.506 (4)	C35—C36	1.381 (8)
C3—C4	1.513 (4)	C35—H35	0.9300
C3—H3	0.9800	C36—H36	0.9300
C4—O1	1.211 (3)	C51—C52	1.359 (4)
C4—C5	1.512 (4)	C51—C56	1.405 (4)
C5—C51	1.518 (4)	C52—C53	1.389 (5)
C5—C6	1.537 (4)	C52—H52	0.9300
C5—H5	0.9800	C53—C54	1.351 (6)
C6—C61	1.510 (4)	C53—H53	0.9300
C6—C7	1.557 (4)	C54—C55	1.361 (5)
C6—H6	0.9800	C54—H54	0.9300
C7—C72	1.438 (5)	C55—C56	1.356 (5)
C7—C71	1.473 (5)	C55—H55	0.9300
C7—H7	0.9800	C56—H56	0.9300
C11—N11	1.132 (4)	C61—C66	1.391 (4)
C12—N12	1.132 (5)	C61—C62	1.401 (4)
C21—C22	1.379 (4)	C62—C63	1.365 (5)
C21—C26	1.385 (4)	C62—H62	0.9300
C22—C23	1.368 (5)	C63—C64	1.359 (6)
C22—H22	0.9300	C63—H63	0.9300
C23—C24	1.343 (7)	C64—C65	1.343 (6)
C23—H23	0.9300	C64—H64	0.9300
C24—C25	1.338 (7)	C65—C66	1.380 (5)
C24—H24	0.9300	C65—H65	0.9300
C25—C26	1.403 (6)	C66—Cl2	1.723 (3)
C25—H25	0.9300	C71—N71	1.123 (4)
C26—Cl1	1.723 (4)	C72—N72	1.119 (4)
C31—C36	1.366 (5)		
			
C12—C1—C11	109.1 (3)	C25—C26—Cl1	119.8 (3)
C12—C1—C2	113.9 (3)	C36—C31—C32	118.6 (4)
C11—C1—C2	110.6 (3)	C36—C31—C3	120.9 (4)
C12—C1—H1	107.7	C32—C31—C3	120.5 (3)
C11—C1—H1	107.7	C33—C32—C31	119.7 (5)
C2—C1—H1	107.7	C33—C32—H32	120.2
C21—C2—C1	112.3 (2)	C31—C32—H32	120.2
C21—C2—C3	112.2 (2)	C34—C33—C32	120.1 (7)
C1—C2—C3	110.6 (2)	C34—C33—H33	120.0
C21—C2—H2	107.1	C32—C33—H33	120.0
C1—C2—H2	107.1	C33—C34—C35	122.1 (7)
C3—C2—H2	107.1	C33—C34—H34	118.9
C31—C3—C4	107.9 (2)	C35—C34—H34	118.9
C31—C3—C2	113.9 (2)	C34—C35—C36	118.5 (8)
C4—C3—C2	110.4 (2)	C34—C35—H35	120.7
C31—C3—H3	108.2	C36—C35—H35	120.7
C4—C3—H3	108.2	C31—C36—C35	120.9 (6)
C2—C3—H3	108.2	C31—C36—H36	119.5
O1—C4—C5	121.6 (3)	C35—C36—H36	119.5
O1—C4—C3	121.8 (2)	C52—C51—C56	118.2 (3)
C5—C4—C3	116.6 (2)	C52—C51—C5	122.1 (3)
C4—C5—C51	108.1 (2)	C56—C51—C5	119.7 (3)
C4—C5—C6	111.2 (2)	C51—C52—C53	121.3 (3)
C51—C5—C6	113.2 (2)	C51—C52—H52	119.4
C4—C5—H5	108.1	C53—C52—H52	119.4
C51—C5—H5	108.1	C54—C53—C52	119.2 (4)
C6—C5—H5	108.1	C54—C53—H53	120.4
C61—C6—C5	114.2 (2)	C52—C53—H53	120.4
C61—C6—C7	111.5 (2)	C53—C54—C55	120.7 (4)
C5—C6—C7	110.0 (2)	C53—C54—H54	119.7
C61—C6—H6	106.9	C55—C54—H54	119.7
C5—C6—H6	106.9	C56—C55—C54	120.8 (3)
C7—C6—H6	106.9	C56—C55—H55	119.6
C72—C7—C71	109.3 (3)	C54—C55—H55	119.6
C72—C7—C6	112.3 (3)	C55—C56—C51	119.9 (3)
C71—C7—C6	112.6 (3)	C55—C56—H56	120.1
C72—C7—H7	107.4	C51—C56—H56	120.1
C71—C7—H7	107.4	C66—C61—C62	116.1 (3)
C6—C7—H7	107.4	C66—C61—C6	122.2 (3)
N11—C11—C1	176.4 (4)	C62—C61—C6	121.7 (3)
N12—C12—C1	178.2 (4)	C63—C62—C61	121.4 (3)
C22—C21—C26	116.4 (3)	C63—C62—H62	119.3
C22—C21—C2	121.7 (3)	C61—C62—H62	119.3
C26—C21—C2	121.9 (3)	C64—C63—C62	120.7 (4)
C23—C22—C21	122.8 (4)	C64—C63—H63	119.6
C23—C22—H22	118.6	C62—C63—H63	119.6
C21—C22—H22	118.6	C65—C64—C63	119.8 (4)
C24—C23—C22	119.8 (4)	C65—C64—H64	120.1
C24—C23—H23	120.1	C63—C64—H64	120.1
C22—C23—H23	120.1	C64—C65—C66	120.8 (4)
C25—C24—C23	120.2 (4)	C64—C65—H65	119.6
C25—C24—H24	119.9	C66—C65—H65	119.6
C23—C24—H24	119.9	C65—C66—C61	121.2 (3)
C24—C25—C26	120.9 (4)	C65—C66—Cl2	117.7 (3)
C24—C25—H25	119.5	C61—C66—Cl2	121.1 (2)
C26—C25—H25	119.5	N71—C71—C7	179.0 (5)
C21—C26—C25	119.9 (4)	N72—C72—C7	179.0 (5)
C21—C26—Cl1	120.3 (3)		
			
C12—C1—C2—C21	−71.0 (3)	C4—C3—C31—C36	121.2 (3)
C11—C1—C2—C21	52.3 (3)	C2—C3—C31—C36	−115.9 (4)
C12—C1—C2—C3	55.2 (3)	C4—C3—C31—C32	−57.2 (4)
C11—C1—C2—C3	178.5 (2)	C2—C3—C31—C32	65.7 (4)
C21—C2—C3—C31	175.7 (3)	C36—C31—C32—C33	1.9 (6)
C1—C2—C3—C31	49.5 (3)	C3—C31—C32—C33	−179.7 (4)
C21—C2—C3—C4	−62.7 (3)	C31—C32—C33—C34	−0.9 (8)
C1—C2—C3—C4	171.1 (2)	C32—C33—C34—C35	−2.0 (12)
C31—C3—C4—O1	92.2 (3)	C33—C34—C35—C36	3.8 (13)
C2—C3—C4—O1	−32.9 (3)	C32—C31—C36—C35	−0.1 (6)
C31—C3—C4—C5	−88.1 (3)	C3—C31—C36—C35	−178.5 (5)
C2—C3—C4—C5	146.9 (2)	C34—C35—C36—C31	−2.7 (10)
O1—C4—C5—C51	95.0 (3)	C4—C5—C51—C52	117.7 (3)
C3—C4—C5—C51	−84.7 (3)	C6—C5—C51—C52	−118.7 (3)
O1—C4—C5—C6	−29.7 (3)	C4—C5—C51—C56	−61.6 (3)
C3—C4—C5—C6	150.5 (2)	C6—C5—C51—C56	62.0 (3)
C4—C5—C6—C61	−58.8 (3)	C56—C51—C52—C53	−0.1 (5)
C51—C5—C6—C61	179.2 (2)	C5—C51—C52—C53	−179.4 (3)
C4—C5—C6—C7	174.9 (2)	C51—C52—C53—C54	−1.1 (6)
C51—C5—C6—C7	53.0 (3)	C52—C53—C54—C55	1.2 (7)
C61—C6—C7—C72	55.6 (3)	C53—C54—C55—C56	−0.1 (7)
C5—C6—C7—C72	−176.6 (3)	C54—C55—C56—C51	−1.2 (6)
C61—C6—C7—C71	−68.4 (3)	C52—C51—C56—C55	1.3 (5)
C5—C6—C7—C71	59.4 (3)	C5—C51—C56—C55	−179.4 (3)
C12—C1—C11—N11	102 (6)	C5—C6—C61—C66	136.4 (3)
C2—C1—C11—N11	−24 (7)	C7—C6—C61—C66	−98.2 (3)
C11—C1—C12—N12	−131 (13)	C5—C6—C61—C62	−46.5 (4)
C2—C1—C12—N12	−7(13)	C7—C6—C61—C62	78.9 (3)
C1—C2—C21—C22	74.6 (4)	C66—C61—C62—C63	−0.2 (5)
C3—C2—C21—C22	−50.7 (4)	C6—C61—C62—C63	−177.5 (3)
C1—C2—C21—C26	−106.2 (3)	C61—C62—C63—C64	0.5 (6)
C3—C2—C21—C26	128.5 (3)	C62—C63—C64—C65	−1.2 (6)
C26—C21—C22—C23	−0.1 (5)	C63—C64—C65—C66	1.8 (7)
C2—C21—C22—C23	179.2 (3)	C64—C65—C66—C61	−1.5 (6)
C21—C22—C23—C24	−1.4 (6)	C64—C65—C66—Cl2	179.2 (3)
C22—C23—C24—C25	1.3 (7)	C62—C61—C66—C65	0.7 (4)
C23—C24—C25—C26	0.1 (7)	C6—C61—C66—C65	178.0 (3)
C22—C21—C26—C25	1.5 (5)	C62—C61—C66—Cl2	180.0 (2)
C2—C21—C26—C25	−177.8 (3)	C6—C61—C66—Cl2	−2.7 (4)
C22—C21—C26—Cl1	−177.3 (3)	C72—C7—C71—N71	174 (100)
C2—C21—C26—Cl1	3.4 (5)	C6—C7—C71—N71	−60 (27)
C24—C25—C26—C21	−1.5 (7)	C71—C7—C72—N72	31 (24)
C24—C25—C26—Cl1	177.2 (4)	C6—C7—C72—N72	−94 (24)

### Hydrogen-bond geometry (Å, °)

**Table:**

*D*—H···*A*	*D*—H	H···*A*	*D*···*A*	*D*—H···*A*
C7—H7···N11^i^	0.98	2.33	3.186 (4)	145
C23—H23···N11^ii^	0.93	2.65	3.409 (5)	139
C34—H34···N72^iii^	0.93	2.52	3.443 (8)	176
C52—H52···N12^iv^	0.93	2.64	3.489 (5)	152
C36—H36···N12^iv^	0.93	2.96	3.805 (7)	152
C54—H54···N71^v^	0.93	2.91	3.564 (6)	128
C53—H53···N71^v^	0.93	2.96	3.583 (5)	126
C64—H64···Cl2^vi^	0.93	2.97	3.663 (5)	133
C64—H64···O1^vi^	0.93	2.88	3.673 (5)	144
C65—H65···Cl1^vi^	0.93	2.80	3.728 (4)	174

Symmetry codes: (i) *x*, −*y*+1/2, *z*−1/2; (ii) −*x*+2, −*y*+1, −*z*+2; (iii) *x*, −*y*−1/2, *z*+1/2; (iv) −*x*+2, −*y*, −*z*+2; (v) −*x*+2, *y*+1/2, −*z*+3/2; (vi) −*x*+1, *y*−1/2, −*z*+3/2.
